# Increasing Rural Recruitment and Retention through Rural Exposure during Undergraduate Training: An Integrative Review

**DOI:** 10.3390/ijerph17176423

**Published:** 2020-09-03

**Authors:** Jens Holst

**Affiliations:** Department of Nursing and Health Sciences, Fulda University of Applied Sciences, Leipziger Strasse 123, D-36037 Fulda, Germany; jens.holst@pg.hs-fulda.de; Tel.: +49-661-9640-6430

**Keywords:** undergraduate medical training, rural exposure, rural placement, rural practice, recruitment and retention, curriculum, medical workforce, integrative review

## Abstract

*Objectives:* Ensuring nationwide access to medical care challenges health systems worldwide. Rural exposure during undergraduate medical training is promising as a means for overcoming the shortage of physicians outside urban areas, but the effectiveness is widely unknown. This integrative review assesses the effects of rural placements during undergraduate medical training on graduates’ likelihood to take up rural practice. *Methods:* The paper presents the results of a longitudinal review of the literature published in PubMed, Embase, Google Scholar and elsewhere on the measurable effects of rural placements and internships during medical training on the number of graduates in rural practice. *Results:* The combined database and hand search identified 38 suitable primary studies with rather heterogeneous interventions, endpoints and results, mostly cross-sectional and control studies. The analysis of the existing evidence exhibited predominantly positive but rather weak correlations between rural placements during undergraduate medical training and later rural practice. Beyond the initial scope, the review underpinned rural upbringing to be the strongest predictor for rural practice. *Conclusions:* This review confirms that rural exposure during undergraduate medical training to contributes to recruitment and retention in nonurban settings. It can play a role within a broader strategy for overcoming the shortage of rural practitioners. Rural placements during medical education turned out to be particularly effective for rural-entry students. Given the increasing funding being directed towards medical schools to produce graduates that will work rurally, more robust high-quality research is needed.

## 1. Introduction

Safeguarding medical care in rural areas is currently posing growing challenges to healthcare systems around the world, including smaller and relatively densely populated countries. Ever-growing problems in maintaining a sufficient level of nationwide medical care, particularly in rural, remote and structurally weak areas, challenge many countries around the world. Even high absolute numbers of physicians available in a country do not prevent it from experiencing the threat of physician shortages in rural and remote regions.

A long catalogue of measures exists to alleviate the shortage of rural practitioners and promote young medical professionals’ motivation to work in rural areas. It ranges from changes in outpatient or inpatient care delivery to structural cross-sector adjustments, including changes in the roles and tasks of other healthcare professions. Other interventions to recruit and retain medical doctors in rural and remote areas, such as financial incentives, targeted and international recruitment, professional and personal support for physicians working in rural and remote areas, and educational interventions, are regarded as important levers for a sustainable increase in the likelihood of future doctors to work in rural areas. The World Health Organization recommends a series of measures to overcome the shortage of rural practitioners: (1) the targeted selection of students with a rural background, (2) study opportunities outside large hospitals and larger cities, (3) clinical placements and internships in rural areas during undergraduate training, and (4) a stronger orientation of curricula towards general medicine contents and topics which are relevant to rural medical practice, ranging from outreach health promotion to the development of practical skills for medical care under resource-poor conditions [1].

Along with elective courses and some isolated initiatives, even high-income countries are still lagging behind in the endeavour to integrate rural health topics into medical education [2]. However, some countries, especially large states such as Canada, Australia and the USA, where the consequences of rural practitioner shortages are much more dramatic due to the enormous geographical distances, have made better progress. Since the 1990s, these countries have pursued various strategies to generate young professionals for rural medical care through adequate interventions during medical training.

Abundant literature points to the potential of appropriate educational interventions in medical training to increase the likelihood of taking up rural practice after graduation. The evidence, however, is to a large extent based on qualitative studies assessing the effect of general and especially rural medical placements or other rural-oriented curricular interventions in medical training programmes on the priority setting and stated future planning of medical students (e.g., [3,4,5,6,7]). In addition, the vast majority of these studies originate from the initiators of rural placement programmes or other curricular interventions aimed at increasing the motivation of medical graduates to practise in rural regions. The mostly positive assessment of the effectiveness of these interventions in medical training on the prospects of success is therefore likely to be subject to a certain bias.

The objective of this integrative longitudinal analysis was to extend the body of evidence regarding the expected positive effect of rural placements and other curricular interventions during undergraduate medical training on the recruitment or retention of rural practitioners. Therefore, the present review assesses the potential of all types of rural-practice interventions in undergraduate medical education to measurably improve the recruitment and retention of rural practitioners. A systematic analysis of existing studies promises to provide evidence on whether a stronger orientation of medical training towards rural medical practice actually and quantifiably contributes to an increase in the recruitment and retention of physicians in rural and remote areas.

While recruitment focuses on attracting physician and medical students to work in rural positions, retention refers to keeping employed health professionals employed in rural practice, the first being an indispensable condition for the latter. As rural employment is the essential outcome of this review, data about recruitment and retention have to be taken into consideration. Measuring the effectiveness of rural internships during undergraduate medical training needs to count both the number of physicians taking up rural practice and of those working in rural and remote areas. Consequently, the review focuses on the geographical locations of physicians after graduation. The intervention consists of a relatively broad array of rural placements during undergraduate training, ranging from short-term assignments to several years of training in rural areas. Medical graduates who have not taken part in any rural training serve as comparison groups. The exclusive outcome of this literature review is the number or share of medical graduates practicing as generalists or specialists in rural or remote areas; mere expressions of desire or intention by medical students or graduates were not taken into account. To obtain a broader picture, no restrictions were made regarding the study design, the medical specialty, the examination year, the country or the region.

This raises the question as to whether, and to what extent, findings in one country can be transferred to others which have different health and educational systems. However, international health systems comparisons often reveal surprising similarities between basic structures and functionalities of healthcare systems, which tend to exhibit common characteristics beyond the specific features and arrangements at first glance. Hence, the analysis of selected country experiences can provide useful information for policy conclusions and recommendations to be applied by other countries. The objective of this review was to extend the body of evidence regarding the positive effect of rural placements and other curricular interventions in undergraduate medical training on the recruitment and retention of rural practitioners.

## 2. Materials and Methods

This paper is based on a literature search aimed at identifying all types of quantitative research in respect to the impact of undergraduate rural medical training interventions on the factual practice of graduates in rural or remote areas. The primary search was carried out between September and November 2018 in the relevant databases PubMed and Embase, using a combination of the terms “medical AND training”, “rural AND health”, “recruitment” and “retention”, combined with the Boolean operators “OR” and “AND”. In order to obtain a longer-term and integrative picture, no timely, technical, geographical or system-related restrictions were made. An additional search was carried out using Google Scholar as well as the unspecific search engine Google with the same combination of terms. Important additions were provided by an analysis of the relevant literature retrieved from the bibliographic references of the identified studies. The following Table 1 exhibits the target group, the type of intervention, control, and the outcomes examined

The indispensable prerequisite for a study to be included in the review was quantifiable information about the type and location of physicians’ practices after graduation. Only quantitative analyses of the relationship between all types of rural curriculum intervention and content and the actual commencement or exercise of medical practice in a nonurban, structurally weak or remote area allowed conclusions to be drawn about the effectiveness of curricular interventions. This review does not include purely qualitative studies and studies concerning the plans and intentions of students expressed in connection with the content of rural medicine training, unless they also contained quantifiable information on rural medical practice.

Likewise, studies on the effects of rural internships and other curricular interventions in medical education on the frequency of subsequent advanced training or practice in general medicine were not included, unless they also contained information on the number of physicians actually practicing in rural and remote areas. This is worth mentioning as specialisation in general medicine is often regarded as a favourable prerequisite or even as a proxy for rural practice, which primarily requires generalists and fewer specialists. Despite the fact that rural general practitioners play a predominant role in many studies, this review did not restrict the scope to generalists and family doctors as some studies also included the effect of nonurban placements on the rural location of specialists.

The initial search with the above-mentioned terms in pertinent databases delivered 276 hits (171 in PubMed, 105 in Embase), of which 61 hits appeared to be primarily suitable according to the headings. In addition, the Google Scholar search and the additional manual search in the unspecific search engine brought 21 more publications to light that had not been detected in the database searches. The review also encompassed repeated direct online searches for papers not included in the literature databases, targeted online searches for additional articles by authors who were frequently identified in the database search, and a review of the bibliographies of the studies and reports retrieved. The screening of the respective reference lists pointed to a further 72 studies that promised to be relevant in answering the research question. After reviewing the titles and abstracts, 49 (31.8%) of the total of 154 publications were excluded as they did not fulfil the study criteria, and another 67 studies were excluded for the same reason after reviewing the full texts (43.5%). The reasons for exclusion were as follows:

- Lack of quantitative information on physicians practicing in rural areas after graduation,
- Restriction to student opinions and declarations of intent,
- Provision of purely qualitative information on various aspects of rural tracks,
- Information on the effectiveness of postgraduate interventions,
- Reference to outcomes other than rural practice,
- Mere expressions of opinions and expert perceptions.

**Figure 1:**
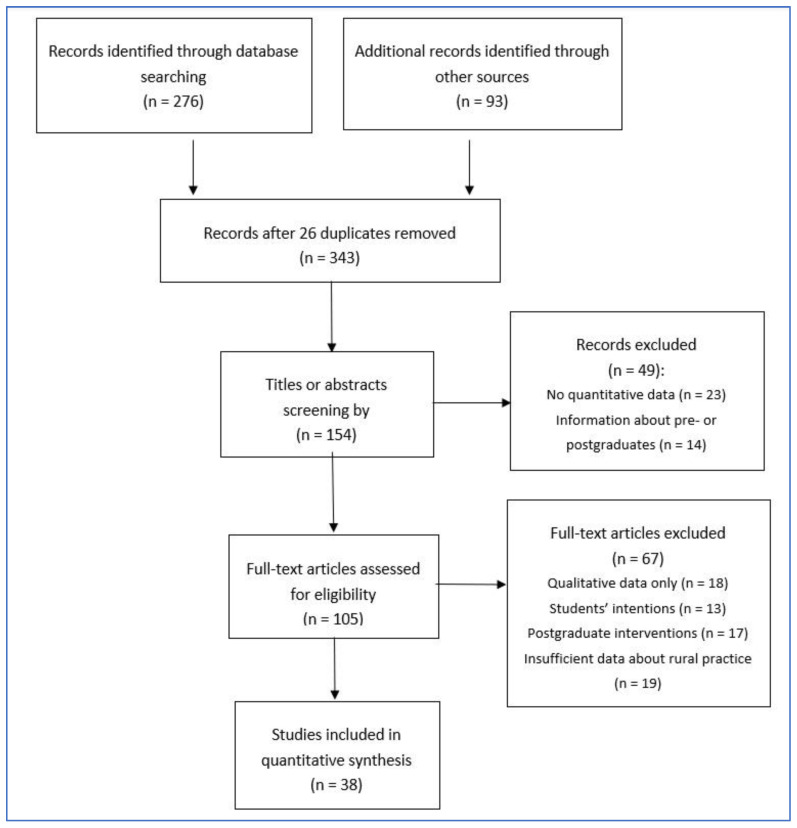
Flow diagram of study selection.

As shown in the flow diagram below (Figure 1), 38 primary studies (16 from the database search, 19 from the review of bibliographies and 3 from the hand search) were finally included in the literature review. The largest number of primary studies, published between 1987 and 2018, stem from Australia (14), followed by the United States of America (13), Canada (7), Norway (2), Japan and the Philippines (1 each). The majority of the hits were published in English, in addition to one French and one Norwegian article, which could be found due to the English abstracts (a detailed presentation of the studies included can be found in Appendix A.

The assessment of the study quality was performed by the author, taking into consideration the clarity of the research question and objective, specification of the study population, consistency of the target population, comprehensibility of the information on inclusion and exclusion criteria, appropriateness of the time frame of the survey, determination of the exposure measures or levels, clarity and reliability of the information on outcome measures and the recording of possible confounders. Due to a general lack of information on the participation rates, strength or variance and effect estimates or dropouts, particularly in older studies, these parameters could not be strictly applied in the comparative quality analysis in order to not lose potentially interesting results.

## 3. Results

All in all, the primary studies exhibit a pronounced heterogeneity, particularly in terms of the size of the study populations and the type and intensity of interventions, ranging from short rural assignments to complete rural medical training in a geographically remote school. Moreover, the objectives associated with curricular interventions in undergraduate medical education and, above all, the endpoints examined differ considerably among the studies included in the review, of which 15 (39.5%) were control studies and cross-sectional studies, respectively; nine were database-driven and six survey-based. Moreover, the review includes five predominantly survey-based longitudinal studies (13.2%), one quasi-experimental study (2.6%) and two mixed-methods studies (5.3%).

A total of 23 of the 38 studies compared the share of rural medical practitioners between medical students who had taken part in some type of rural internship during undergraduate training and those who had not participated in such interventions. Rural exposure during medical education increased the likelihood of later rural practice by more than four times (4.2) on average, but with a wide variation ranging between 1.34 and 19.1 (standard deviation 3.92) [8,9,10,11,12,13,14,15,16,17,18,19,20,21,22,23,24,25,26,27,28].

One study also investigated the effect of interventions in different phases of medical education and found rural internships and tracks towards the end of undergraduate training to be particularly effective (the likelihood of later rural practice increased by 1.5 after internships in the third year, and by 3.0 in the fifth year) [29]. Another analysis showed a clear correlation of postgraduate rural practice with the length of medical training in a rural environment: Compared to 1 to 2 years, 3 to 4 years of training in rural and remote medical schools increased the likelihood of becoming a rural practitioner by 9.3 instead of 2.5 times [30]. Thus, despite the variability of the findings, it can be assumed that rural curriculum interventions in undergraduate medical training have the potential to favour later medical practice in rural and remote areas; this correlation is rather weak for some interventions but relatively strong for others.

Ten studies recorded the share of rural practitioners after medical training outside larger cities and urban medical schools. The results were also quite heterogeneous, because three studies showed a share of less than 50% [31,32,33], five studies slightly above 50% [34,35,36,37,38,39], one study did not explicitly quantify the proportion [40], and only one study exhibited a very large share of medical graduates whose undergraduate training included rural practice interventions and who took up jobs in rural areas [41]. The average share was 51.3%, with a standard deviation of 17.5%. Despite the heterogeneity of the results, the proportion of rural practitioners following targeted curricular interventions tends to be higher than after conventional medical education.

Five studies determined the probability of taking up rural practice after tracks, internships and other training opportunities outside cities and urban medical schools, mainly by means of odds ratios, which were 2.12 (*p* = 0.15) [42], 2.6 (95% CI = 2.1–3.4) [43], 6.4 [44] and 8.4 (95% CI = 2.1–33.5, *p* = 0.002) [32], or the prevalence odds ratio, which was 3.9 [45]. Here, too, the obvious variability of the results has to be stressed. A further study assessed the effectiveness of rural exposure during undergraduate medical training in relation to urban or rural origin of students and found that an internship outside larger cities can even outweigh the aversion of urban-entry students to rural practice [46].

While two of the studies provided evidence for an intuitively comprehensible dose-response relationship [47] between the length and intensity of rural exposure during undergraduate medical training and the likelihood to work as rural practitioner [29,30], the empirical basis for this assumption remains rather weak. A number of studies provide evidence that, in particular, longer internships and rotations during medical training correlate with positive rural workforce outcomes [11,30,32,48]. It should be noted, however, that longer periods of rural exposure during undergraduate training were often followed by additional rural training and postgraduate experience. This makes it impossible to separate the positive correlation properly from the effects of postgraduate rural practice [49].

## 4. Discussion

Overcoming the growing medical workforce shortages in rural areas of many countries worldwide requires effective and sustainable strategies. Increasing the number of medical practitioners in rural and remote areas through medical training interventions is a complex health-policy challenge that calls for a broad range of intelligent and coordinated measures. Universities and medical schools can make a significant contribution.

One step would be the further propagation and upgrading of academic general practice and family medicine, particularly in those countries where generalists do not have the same reputation as other clinical specialists [1]. In addition to a more practical orientation of undergraduate medical education, strengthening general medicine is considered a relevant prerequisite for improving healthcare in rural areas [50]. This review shows that the combination of the two approaches is promising, particularly a stronger practical orientation in the form of rural tracks, which in almost all included studies focused on general and family medicine. Extending the availability and intensity of rural exposure in undergraduate medical education ultimately requires general medicine chairs to receive the same rights, possibilities and perspectives as their colleagues in other clinical disciplines. In order to make general medicine more practice-oriented and thereby more attractive, providing care to patients must be or become an important part of the scope of tasks in academic general medicine. This will hardly work as long as general and family medicine is not a part of the standard healthcare provided by academic medical schools, and as long as university general practice lecturers cannot bill for medical services in the same way as their colleagues in other specialisation fields.

This review identified and assessed a critical number of studies on the effectiveness of rural exposure during undergraduate medical training in increasing the rural workforce. In particular, longer and more intensive rural placements of medical students turned out to be promising in contributing to a moderate or even significant increase in the number of graduates working in rural practice. This assessment is consistent with other meta-analyses and reviews [50,51,52,53] showing that particularly larger programmes, which combine several approaches and measures, lead to a higher share of medical graduates taking up employment in rural areas [54,55].

The strongest effect can be observed if a relevant part or the whole medical training take place at a rural or remote medical school, i.e., outside metropolitan and large cities. However, findings are inconsistent here as well [51,56,57], which is probably due, among other things, to the diversity of the interventions. Understandably, the outcome of rural placements and tracks during medical training depends not only on the extent, but also on the nature and quality of rural exposure [58]. Despite this, some findings question the connection between positive experiences of students acquired during rural medical placements or internships and subsequent rural practice [59]. Further research is needed to determine whether and under what conditions longer or more intensive internships and rotations during undergraduate medical education can effectively contribute to increasing the rural medical workforce.

Beyond the actual research question, this review revealed another finding, which is worth mentioning in this context as it is highly important for the evaluation of the effectiveness of rural interventions and exposures during medical training: the most promising strategy for promoting employment in rural practice after graduation seems to be the targeted selection of students of rural origin who are more likely than others to end up working in rural areas. A considerable share of studies included in this review show that the rural upbringing of medical students or their partners turns out to be the strongest predictor for rural medical practice [13,16,17,18,20,21,23,26,28,37,38,41,42,43]. This finding is consistent with a large number of earlier observations and findings [1,52,55,57,60,61,62,63,64]. Physicians who have grown up or at least spent a certain time of their lives in the countryside or in small towns are much more likely to take up a rural practice than their colleagues with an urban upbringing. In addition, rural medical training tracks and internships had the greatest effect on future medical students with a rural background [21].

Hence, prioritising the allocation of medical school places to students of rural origin might be a promising approach for training physicians who are willing to take up rural medical practice after graduation. It can be assumed that an appropriate adaptation of the framework conditions and admission criteria for medical school assignments can increase the effectiveness of internships in rural medicine and thereby contribute to reducing the undersupply of rural medical workforce. However, such a policy has to take into account the trade-off between individual freedom and regulatory interventions, and it might create a series of concerns regarding fairness, individual priority setting and confidentiality of data, among others. Moreover, the inclusion of voluntary or possibly mandatory rural exposure, i.e., outside university clinics, teaching hospitals and urban practices, in the curricula of medical schools can contribute to an increase in the share of graduates taking up rural medical practice [65,66]. The suspicion that more and prolonged rural internships and placements in undergraduate medical training could negatively affect the level of performance proves to be unfounded [15,41,67].

## 5. Limitations

The present analysis of the existing evidence regarding the impact of rural interventions during undergraduate medical training on the recruitment and retention of rural practitioners has several limitations. First of all, the availability of studies with good and reliable empirical evidence is rather low. Although some studies of higher quality have been published in recent years, the evidence for rural exposure during undergraduate medical training having contributed to overcoming the shortage of rural practitioners is relatively weak. Another fundamental limitation of this review results from the conceptual and methodological heterogeneity of many primary studies included, which exhibit important differences with regard to both the interventions and endpoints. Hence, a summary assessment of the existing evidence was only possible according to the quite-general criteria and without sufficient consideration of any special features in the respective countries and health systems.

Moreover, only a small number of studies systematically consider potential confounders that may have influenced the decision of graduates to take up rural practice independently of the intervention observed in the respective study. These may be individual factors such as rural upbringing and family or social ties, framework conditions such as additional incentives, for example in the form of grants and special remuneration opportunities, support in setting up and leading practices, or special training opportunities.

It has to be mentioned that this review does not address the difference that exists between recruitment and retention, although the barriers to long-term employment are certainly higher than those for taking up rural practice. This limitation derives from the fact that the studies identified did not exhibit differentiated data regarding recruitment and retention—they captured rural practice at a given point in time, and not its duration. Hence, it was impossible to draw specific conclusions about both rural recruitment and retention.

Finally, one should bear in mind that the results from one country cannot be simply transferred to other countries. Different training, financing and healthcare systems coincide with other incentive and control measures to increase the share of rural physicians, and different (health) policy frameworks limit the generalisability of the findings described above. However, due to the fundamentally similar trend of the study results, regardless of region, system and other influencing factors, the transferability of some country-specific findings can be assumed [52,68]. Different framework conditions regarding the centralisation or decentralisation of higher education and country-specific political, normative, financial and administrative arrangements have rather indirect effects on the relationship between curricular interventions and outcomes. Moreover, they do not call into question the general relationships presented in the review, especially as the impedimental causes for increasing the medical workforce are in principle very similar in all countries [1,63,69].

## 6. Conclusions

The present review contributes to the existing literature as it provides a longitudinal overview of the outcome of a broad array of attempts and approaches to promote rural practice through interventions in undergraduate medical training in a number of countries around the world. The global focus does not only reconfirm the potential of rural exposure during undergraduate medical training in contributing to recruitment and thereby also to retention in nonurban settings, it also shows the generally increasing effectiveness of such interventions over time. Regardless of the country and healthcare system in place, rural placements during medical training have some potential to support the endeavour to reduce the shortage of rural physicians. Along with the control of supply and demand, financial incentives and other measures, undergraduate medical education can play a considerable role in the recruitment and later retention of rural physicians. Beyond the underlying research question in the narrower sense, the review corroborates existing evidence for the preferential admission of students with a rural upbringing to medical schools as being an important component of a successful strategy to maintain and stabilise the medical workforce in nonurban and even remote regions. Medical education outside larger cities and highly specialised university hospitals, which familiarises students with special features of rural healthcare, is promising to promote graduates to take up rural practice—this applies particularly to rural-entry students. In addition, more intensive, mandatory contact with healthcare outside larger cities and metropolitan areas can contribute to the overall upgrade of general practice and family medicine, and especially rural medicine. There is a need for further research to clarify which interventions are most successful in increasing the likelihood of prospective medical graduates to work in rural practices, and which additional factors tend to be promotive or inhibitory for rural practice.

## Figures and Tables

**Table 1:** PICO scheme.

**P**opulation	Medical students
**I**ntervention	All types of rural placements and internships during undergraduate training
**C**omparator	No rural exposure during undergraduate training
**O**utcome	Rural employment after graduation

## Appendix A

**Study**	**Year**	**Location**	**Study Type**	**Intervention Type**	**Target Group**	**Students (n)**	**Outcomes**	**Findings**	**Specificity**	**Quality**
May et al. [26]	2018	New South Wales/Australia	Retrospective cross-sectional cohort study	Extended Rural Clinical School exposure: semester- or year-long rural placement in year 4 and 5	Domestic medical students graduated between 2012 and 2014	171 426 (out of 435 = 98%)	Practice location in under-served district	Rural placement ≥1 year multiplies chance to practice in rural location 3.5 years after graduation by six (OR 6.075, 95%-CI 2.716–13.591)	Fair	+
Woolley et al. [28]	2017	Philippines	Data-based retrospective cohort control study	1 month per semester in community placements across years 1–3, followed by 10 months of community placement in year 4	Medical students at Ateneo de Zamboanga University and University of the Philippines in Manila	232 graduates in Zamboanga, 121 graduates in Manila. Control cohort: 464 and 264 graduates in medical schools with more conventional curricula	Medical practice in communities <100,000 population; practice in lower-income communities	Practice in smaller communities: - 31% of exposed graduates vs. 7% of graduates from the conventional school in Zamboanga (*p* < 0.001) - 61% of graduates vs. 12% of graduates from the conventional school in Manila (*p* < 0.001)	Fair: Low identification rate of conventionally trained graduates	+–
Wheat et al. [25]	2017	Alabama/USA	Quasi-experimental nonrandomised control study	Rural Medical School training (as part of the Rural Medical Scholars Programme)	Medical students enrolled between 1997–2002	54 rural vs. 182 regional and 649 main campus students	Production of rural physicians	Share of rural practitioners: - Rural graduates: 48.1% (OR 6,4, *p* < 0.001) - Regional campuses: 23.8% (OR 2,5, *p* < 0.001) - Main campus: 11.2% (OR 1.0)	Fair	+
Nelson et al. [44]	2017	Iowa/USA	Data-based retrospective cohort study	Iowa Family Medicine Training Network	Graduates from Iowa medical schools	1645 (out of 1676) graduates = 98.2%	Rural location decision and 5-year retention	Likelihood of retention strongly related to undergraduate medical training in Iowa (OR = 6.74. *p* < 0.001)	Fair	+
Wendling et al. [24]	2016	Michigan/USA	Retrospective cohort control study	Rural Physician Programme curriculum	Graduates from Michigan University Medical School	152 rural programme graduates vs. 2230 nonrural graduates	Primary healthcare practice in rural area (acc. to Rural-Urban Commuting Area Code, RUCA)	Rural-programme students more likely than others to practice in - Rural areas (76/168 [45%] vs. 361/2610 [14%]; *p* < 0.001) - Health Professional Shortage Areas (106/168 [63%] vs. 1279/2610 [49%]; *p* < 0.001)	Fair	+
Kondalsamy-Chennakesavan et al. [23]	2015	Queensland/Australia	Retrospective comparative cohort study	Undergraduate medical training at Rural Clinical School	Medical students	754 (out of 1572 = 48%): 236 (31.3%) had a rural background, 276 (36.6%) had attended the University of Queensland (UQRCS).	Current clinical practice in a rural location	Rural clinical practice location: 41.7% (115/276 of Queensland-University attendees) vs. 18.8% (90/478 UQ metropolitan clinical school attendees), *p* < 0.001. Independent predictors of rural practice: (OR [95%-CI]): Queensland University attendance for 1 year (OR 1.84 [1.21–2.82]) or 2 years (2.71 [1.65–4.45]) Independent predictors of rural practice for interaction between UQRCS attendance and rural background: rural background + UQRCS attendance for 1 year (OR4.44 [2.38–8.29]) or 2 years (7.09 [3.57–14.10])Effects of rural background and UQRCS attendance were duration-dependent.	Fair	+
Shires et al. [22]	2015	Tasmania/Australia	Comparative data-based cohort study	≥1 year at Rural Clinical School in smaller city	Medical students in larger cities and at least 1 year in smaller cities	974 larger-city graduates vs. 202 RCS graduates	Practice location	Rural-clinical-school graduates 5 times as likely to be working in remote areas than control group: 28% vs. 7%, χ2 = 59.5, *p* < 0.0001 (OR 4.9, 95%-CI 3.2–7.6); Monash model: Nine-fold likelihood (OR 9.0, 95%-CI 4.7–17.2)	Fair: not checked for selection bias	+
Playford et al. [21]	2014	Western Australia	Retrospective control cohort study, logistic regression	≥1 year at Rural Clinical School (RCS)	Medical students at University of Western Australia	1017 graduates (out of 1116 = 91%)	Rural vs. urban practice location	Participation in RCSWA strongly associated with greater likelihood of rural practice: - 14.9% of RCSWA graduates from rural backgrounds vs. 3.8% from urban background controls working rurally - 20.6% of rural-background RCSWA graduates vs. 14.7% of rural-background non-RCSWA controls working rurally	Fair	+
Jamar et al. [33]	2014	Southern Australia	Cross-sectional retrospective cohort study based on a 28-question online survey	Rural 5^th^ year during 6-year undergraduate medical program	Former medical students at University of Adelaide	74 out of 124 (response rate 58.2%)	Practice location in rural area	20.8%–34.1% of respondents located in a rural area; - >50% had spent time in a rural area since graduation	Low	–
Woolley et al. [45]	2014	Queensland/Australia	Multiple logistic regression analysis using data of a longitudinal cohort study	Medical training at a school in regional area	Medical graduates	260 (out of 264, response rate 98%)	Practice in a rural town	Prediction by - Internship in a small rural location (prevalence odds ratios (POR) = 3.9, *p* < 0.001)	Low	+–
Gupta et al. [46]	2014	Northern Queensland/Australia	Cross-sectional survey via email and telephone communication, and via JCU School of Medicine Facebook	Internship in rural/remote areas	Postgraduate interns across postgraduate years 1 to 7	530 (out of 536 = 99%)	Association of later practice location with hometown and internship location	Important association between remoteness area of internship and subsequent rural practice: Likelihood to practice in rural locations: Metropolitan hometown + nonmetropolitan internship > nonmetropolitan hometown + metropolitan internship (OR 6.1 vs. 2.6, *p* < 0.001) and in inner regional locations (OR 3.1 vs. 1.6, *p* = 0.003).	High within the selected group	++
Forster et al. [32]	2013	New South Wales/Australia (UNSW)	Cross-sectional retrospective cohort study based on online survey	1–3 years of undergraduate training at rural clinical school	Medical graduates	214 (out of 315 = response rate 68%)	Current, preferred current and intended career rural locations	26% working in rural area Nonrural medicine entry graduates with 3 years at rural campuses more likely to take up rural practice compared to those with 1 year at a rural campus (OR = 8.4, 95%-CI = 2.1–33.5, *p* = 0.002). OR not significant for 2 vs. 1 year	Low – repeated mix up of current, preferred current and intended rural practice locations	+–
Strasser et al. [41]	2013	Northern Ontario/Canada	Mixed methods studies to assess socioeconomic impact of medical school through post-graduate tracking	Northern Ontario School of Medicine Comprehensive Community Clerkship	Medical students	66	Practice location	61% of all graduates have chosen predominantly rural family-practice training. 65% of the graduates of the Family Medicine programme practice in Northern Ontario or other rural communities	Low: large overlapping with recruitment strategies	+
Eley et al. [31]	2012	Queensland/Australia	Longitudinal sequential study via email survey	Undergraduate Rural Clinical School training	Year 4 students	115 out of 183 = 64%	Practice location	Altogether, 40% working in nonurban locations – high variation (range 0–100%)	Low	+/–
Tate and Aoki [20]	2012	Manitoba/Canada	Retrospective cross-sectional survey (per mail)	Rural medical education experiences during under- (and postgraduate) medical training	Medical graduates of the University of Manitoba in Winnipeg	1269 out of 2578 (=49%)	Rural physician practice	Significant association with rural educational exposure during medical school (and residency training) (*p* = 0.0068): graduates with (partly) undergraduate training in rural settings 1.34 times (95%-CI 1.09 to 1.75) more likely to practise in rural location. Increasing time spent in rural training related to increased likelihood of rural practice	High	+
Whitford et al. [43]	2012	South Australia	Survey based on indirect, mixed mode of recruitment and a questionnaire (South Australian Allied Health Workforce (SAAHW) survey)	Nonmetropolitan university or college and/or had rural placement	Allied health professionals	1539 (82% male, 75% in urban practice) (representing 18.3% of potential target group)	Numerous (incl. rural vs. urban placement)	Practice in a rural location influenced by rural experience during training (χ^2^ [1, n = 1466] = 68.6 [*p* < 0.001], with an OR = 2.6 [95%-CI = 2.1–3.4])	Low: factors influencing rural placement represent only a minor part of the survey	+
Landry et al. [30]	2011	New Brunswick/Québec/Canada	Questionnaire-based survey (reply by telephone or in writing) + multivariate logistic regressions	Exposure to undergraduate medical training in area of interest for, 1, 2, 3, and 4 years; postgraduate exposure	Francophone physicians admitted to Quebec/New Brunswick	263 (out of 390 = response rate 67%); 174 family and 100 specialty physicians	Current practice in intervention region – recruitment and retention	Undergraduate exposure: Relative likelihood to practise in the area according to years of exposure: Family physicians: - 1 year: 2.5 (95%-CI 0.8–7.4); - 2 years: 2.5 (95%-CI 0.7–8.6) - 3 years: 9.3 (95%-CI 1.5–56.9); - 4 years: 9.3 (95%-CI 1.4–60.1). Specialists: No effect of exposure on location of practice.	High	+
Quinn et al. [36]	2011	Minnesota/USA	Comparative retrospective cohort study	Six-month Rural Track Clerkship	3^rd^-year medical students graduated 1997–2009	253	Practice location after graduation	Rural-track participants: - >57% chose rural location for first practice	Low	+/–
Rabinowitz et al. [12]	2011	Pennsylvania/USA	Case-control study based on longitudinal study	Physician Shortage Area Programme	Jefferson Medical College graduates practising rural family medicine and in Pennsylvania’s rural counties	2394	Practice location and specialty	Clearly increased likelihood of Physician shortage are programme graduates to: - Rural family medicine practice (32.0% [31/97] vs. 3.2% [65/2004]; RR = 9.9, CI 6.8–14.4, *p* = 0.001)	Fair	+
Zink et al. [39]	2010	Minnesota/USA	Comparative longitudinal survey based on student and practice data	Rural Physician Associate Programme + focus on recruiting students who will be rural family physicians	Medical school graduates	3365 graduates: 491 intervention group + 2874 control group	Primary care specialisation and rural practice	Students of the two intervention groups most likely to select rural location: 86% vs. 57%; rural practice rate also higher among nonprimary-care specialists graduated from one intervention programme: 43% vs. 15, 11 and 8%.	Fair: Unclear consideration of selection bias	+
Straume and Shaw [27]	2010	Finnmark/North Norway	Longitudinal survey based on graduates’ data	Intern support project – mandatory internship in a very remote region with group tutorial and day-to-day supervision	Interns	233 (out of 267, response rate 87.3%), 112 men and 121 women; 18.5% Northern Norway origin, 150 (64.4%) from the South, 40 from abroad	Rural practice in Northern Norway after graduation	Increased likelihood to choose both primary healthcare and a job in remote Northern Norway: 45.9% took first job in Finnmark – highly significant difference (*p* < 0.001) compared to geographical origin; 89% of graduates from the north took first job there – 8 times as likely compared to those from the south	Fair – large overlapping with origin	+
Bustinza et al. [42]	2009	Lower St. Lawrence Region, Québec/Canada	Mail-based cross-sectional questionnaire survey and data-based survey (Bas-Saint-Laurent Regional Department of Health and Social Services), Cox proportional hazards model	(Cumulative duration of) Rural rotations in the region	Family physicians practising in the area between 1985 and 2003	215 (out of 644 = 33% return rate)	Rural practice and retention	Adjusted probability of physicians remaining in Bas-Saint-Laurent after being exposed to the area through rural rotations had an odds ratio of 2.12 (*p* = 0.15).	Overlapping/selection bias: probability to remain in the area for physicians originally from the region: 4.5 (*p* < 0.01).	+
Jarman et al. [18]	2009	Wisconsin/USA	Retrospective cross-sectional survey based on 51-item online and paper questionnaire on background, interests, location and factors influencing practice choice	Rural clerkship during medical school	Surgery residency programme graduates	84 (out of 216 = 39%; raw return rate 45%)	Urban or rural surgical practice	Likelihood to choose rural practice positively associated to rural clerkship (79% vs. 37%, *p* = 0.001) and surgical residency programme committed to rural training (*p* = 0.046)	Very low: Many confounding qualitative data included in the study	– Very unspecific, rather arbitrary approach
Stagg et al. [38]	2009	Australia	Retrospective, questionnaire-based survey by telephone or online	Parallel Rural Community Curriculum	Medical graduates	46 (out of 86 = 53%)	Rural career path	54% on rural career path - Significant relationship to decision prior to or during undergraduate training (*p* = 0.027); - Graduates in family medicine training more likely to be on rural career pathway than those in other specialties (*p* = 0.003)	Fair	–
Mathews et al. [17]	2008	Memorial University of Newfoundland, Canada	Cross-sectional study based on the Southam Medical database	Medical training at rural medical school	Physicians practising in Canada	1322 out of 1381	Medical practice location in rural Newfoundland and Labrador	167 (12.6%) rural-school graduates working in rural Canada vs. 81 (6.1%) working in rural Newfoundland. Likelihood to practise in rural Canada compared with graduation from urban backgrounds, no residency training at rural medical school or specialists, increased with: - Rural background (OR 1.95, 95%-CI 1.38–2.76) Likelihood to practise in rural Newfoundland compared with graduation from urban backgrounds, no residency training at rural medical school, specialists and non-Newfoundlanders, increased with: - Rural background (OR 2.54, 95%-CI 1.57–4.11), - Upbringing in Newfoundland (OR 7.01, 95%-CI 2.16–22.71)	Weak:Very broad and general intervention, strong selection bias	+
Matsumoto et al. [16]	2008	Japan	Retrospective cohort study	Medical training at Jichi Medical School	Graduates from Jichi Medical School	2988 (follow-up rates 98.7%, 98.2%, and 98.0%, respectively; contract fulfilment 100% for all years)	Workforce outcomes during and after contract fulfilment	Likelihood to work in rural areas of rural-training graduates 4.2 times higher than nonrural graduates.After-duty graduates 3.4 times more likely to work in low population density areas, 2.6 times more likely to work in high elderly rate area, and 2.3 times more likely to work in low physician/population ratio areas;Under-duty rural-programme graduates 8.9 times, 6.5 times, and 5.0 times more likely, respectively	Fair	++
Worley [15]	2008	Adelaide-Alice Springs – Darwin/Australia	Longitudinal comparative retrospective study based on postal survey	Flinders Parallel Rural Community Curriculum: 2-year problem-based learning + decentralised placement in rural areas during 3^rd^ year of undergraduate training	Graduates of Flinders University School of Medicine	13 (out of 28 = response rate 46%)	Workforce outcomes 5 years after graduation	70% practising in rural communities, compared to 18% of tertiary-hospital-trained students	Very low: tiny sample size, no consideration of other factors such as pre-selection and potential biases	+–
Worley et al. [14]	2008	Darwin/Australia	Longitudinal retrospective study based on postal survey	Parallel Rural Community Curriculum at Flinders University+ Northern Territory Clinical School	Graduates of rural and urban Flinders University School of Medicine and of Northern Territory Clinical School	74 (out of 150 = response rate 49%)	Preference for rural vs. urban practice >5 years	Rural training graduates from Flinders 19.1 (95%-CI, 3.4–106.3; *p* < 0.001) and from Northern Territories 4.3 (95%-CI, 1.2–14.8; *p* = 0.026) times more likely to choose rural career paths than graduates from urban Flinders Medical Centre, after adjusting for age and rural background	Good	+
Daniels et al. [13]	2007	New Mexico/USA	Case control study based on a longitudinal data survey and mailed questionnaire	Rural Health Interdisciplinary Programme (elective programme including rural practice)	Graduates from 12 health-professional programmes	765 (out of 1396: 59% return rate + 55 exclusion for incomplete graduation)	First and any rural employment after graduation	Rural practicum completion associated with rural practice choice (*p* < 0.01): - 30% vs. 16% for first employment - 42% vs. 25% for any employment	Low: Overlapping of several partly interlinked factors (rural background, proximity to family)	Fair
Smucny et al. [12]	2005	State University of New York; Upstate Medical University	Mailed, questionnaire-based retrospective survey	Rural Medical Education Program: 36-week clinical experience in rural communities for medical students	Medical students at State University identified acc. to the Physician Masterfiles	76 (out of 132 = 58%)	Geographic practice location	Practice in rural locations < 50,000 people: - 26% (22 out of 86) former rural-education students vs. 7% (95/1307) nonrural education programme students (*p* < 0.0001) - Including residents: 17% (23/132) vs. 6% (112/1969) (*p* < 0.0001)	Fair	+
McCready et al. [11]	2004	Northwest Ontario/Canada	Retrospective data-based control study (SPSS) using the Canadian Medical Directory	Northwestern Ontario Medical Programme(NOMP)	Former NOMP medical students	1982 (out of 2335 NOMP participants = 84.9%)	Practice in Northern Ontario	Undergraduate medical students (and postgraduate residents) with NOMP placement 7.11 more likely to practise in North-Western Ontario (*p* < 0.001, OR 7.11, 95%-CI 5.11–9.90);Single placement (≤1 month): 50/1042 (4.8%) practised in North Western Ontario; longer placement periods usually associated with higher rates of practice (*p* = 0.003)	Fair	+
Alexandersen et al. [37]	2004	Tromsø/Norway	Controlled retrospective study based on data from the Norwegian physician registration file	Medical training at Tromsø University vs. Oslo University	Medical students	Intervention group: 318 out of 345 (=92.3%)Control group: 851 out of 992 (=85.8%	Employment in (rural and remote) North Norway	Recruitment/retention rate of intervention group in North Norway: 51.3% compared to 7.5% of North Norwegians trained in Oslo	Low since the intervention is restricted to regional assignment of university places; relevant selection bias	+
Phillips et al. [10]	1999	Washington/USA	Retrospective cohort study based on data of the Physician Masterfile of the American Medical Association	Family physician curricular pathway	Medical Students at University of Washington	239	Family physicians vs. other pathways	Likelihood to practice in very rural areas fivefold compared to earlier graduation cohorts = non-FP pathway.21 graduates from the intervention groups (3.5%) were rural family physician, more than 10 (10.6) times the 2 graduates (0.33%) from 8 years prior to intervention	Week (very low numbers of control group, no confounders or selection effects taken into consideration)	++
Whiteside and Mathias [35]	1996	British Columbia/Canada	Control study based on mailed cross-sectional survey	Rural training programme of the University of British Columbia	Residents graduated from rural training programme (RR) vs. nonprogramme-trained rural family physicians (RP)	39 RRs (out of 46 = 84.8%) + 35 (out of 46 = 76.1%)	Practice location	51% located in rural areas, 20.5% in regional settings and 17.9% in metropolitan areas.	Fair	+
Rolfe et al. [29]	1995	Newcastle/Australia	Cross-sectional survey based on mailed questionnaire	Rural secondment in year 3.Rural general-practice rotation year 5	Graduates with respective urban practical training	217 (out of 331 medical doctors = 65.6%)	Rural practice	Rural family practice rotation: Rural vs. urban location: 30% vs. 10% (*p* = 0.007, RR 3.02, 95%-CI 1.25–7.32)Rural vs. urban 3^rd^-year secondment: 23% vs. 34% working in rural areas (*p* = 0.26, RR 0.67, 95%-CI 0.37–1.22)	Fair	+
Fryer [8]	1993	Colorado/USA	Control study based on retrospective data analysis (AMA Physician File)	Clinical rotation outside metropolitan area;clinical family practice	UCSM graduates	Total 284.Intervention group: 131 with, control: 153 without ≥ 1 clerkship outside metropolitan areas	Practice in rural counties in Colorado and particularly in towns with <5000 inhabitants	Graduates with nonmetropolitan clerkship in rural practice: 13.7% vs. 7.8% of control group.Practice in small town (<5000): 9.9% vs. 4.0% (*p* = 0.04)	Low	+/–
Verby [34]	1988	Minnesota/USA	Data-based longitudinal cohort study	Rural Physician Associate Programme of 9–12 months	3rd year medical students	327 practising physicians out of 462 RPAP participants	Medical practice location	57% of former rural-programme students in rural practice, mostly in Minnesota and in communities <10,000 inhabitants, compared to estimated 80% for 1971–1975	Very low—no other determinant taken into consideration	+
Adkins et al. [8]	1987	Washington, Alaska, Montana, Idaho/USA	Data-based longitudinal pre-post control study	Cooperative medical education programme	Medical graduates	1757 pre- and 947 postgraduates: 270 without, 677 with cooperative experience, 299 cooperative-programme students who studied the 1st year elsewhere	Geographic and specialty distribution before and after	Higher likelihood of cooperative-programme graduates to practise in rural settings (23% vs. 12%) (no statistical significance reported)	Fair	+
